# Terahertz Spectroscopy Sheds Light on Real‐Time Exchange Kinetics Occurring through Plasma Membrane during Photodynamic Therapy Treatment

**DOI:** 10.1002/advs.202300589

**Published:** 2023-04-25

**Authors:** Xiujun Zheng, Blandine Lordon, Anne‐Françoise Mingotaud, Patricia Vicendo, Rachel Brival, Isabelle Fourquaux, Laure Gibot, Guilhem Gallot

**Affiliations:** ^1^ Laboratoire d'Optique et Biosciences Ecole Polytechnique CNRS INSERM IP Paris Palaiseau 91128 France; ^2^ Laboratoire des IMRCP Université de Toulouse CNRS UMR 5623 Université Toulouse III ‐ Paul Sabatier 118 Rte de Narbonne Toulouse 31062 France; ^3^ Centre de Microscopie Electronique Appliquée à la Biologie Faculté de Médecine Toulouse Rangueil Université de Toulouse 133 route de Narbonne Toulouse 31062 France

**Keywords:** permeability, photodynamic therapy PDT, plasma membrane, polymers, self‐assembly

## Abstract

Methods to follow in real time complex processes occurring along living cell membranes such as cell permeabilization are rare. Here, the terahertz spectroscopy reveals early events in plasma membrane alteration generated during photodynamic therapy (PDT) protocol, events which are not observable in any other conventional biological techniques performed in parallel as comparison. Photodynamic process is examined in Madin‐Darby canine kidney cells using Pheophorbide (Pheo) photosensitizer alone or alternatively encapsulated in poly(ethylene oxide)‐*block*‐poly(*ε*‐caprolactone) micelles for drug delivery purpose. Terahertz spectroscopy (THz) reveals that plasma membrane permeabilization starts simultaneously with illumination and is stronger when photosensitizer is encapsulated. In parallel, the exchange of biological species is assessed. Over several hours, this conventional approach demonstrates significant differences between free and encapsulated Pheo, the latter leading to high penetration of propidium iodide, Na^+^ and Ca^2+^ ions, and a high level of leakage of K^+^, ATP, and lactate dehydrogenase. THz spectroscopy provides, in a single measurement, the relative number of defects per membrane surface created after PDT, which is not achieved by any other method, providing early, sensitive real‐time information. THz spectroscopy is therefore a promising technique and can be applied to any biological topic requiring the examination of short‐term plasma membrane permeabilization.

## Graphical abstract

THz spectroscopy provides, in a single measurement, the relative number of defects per membrane surface created by oxidative stress generated during photodynamic therapy, offering early, sensitive real‐time information. THz spectroscopy is therefore a complementary technique to established (biological) assays and can be applied to any topic requiring the real‐time examination of short‐term plasma membrane permeabilization.

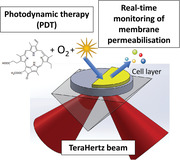

## 1 Introduction

In the electromagnetic spectrum, the terahertz (THz) domain ranging between 0.3 and 10 THz (wavenumbers between 10 and 333 cm^−1^ or wavelengths between 1 mm and 30 µm) has traditionally remained one of the least explored. The main reason is due to the difficulty of providing easy access to reliable THz sources. With the progress made in the last 20 years, this drawback is now being overcome. Consequently, THz spectroscopy is being increasingly assessed in applications spanning from security in airports to medical imaging. THz spectroscopy has been shown to be sensitive to collective associations between molecules, an asset in the field of soft matter. Beyond application in the materials domain, the THz region of the electromagnetic spectrum has significant potential in the biomedical field.^[^ ^1^ ^]^ The interaction between THz radiation and the vibrational modes of biological liquids enables the development of original biological diagnostic sensors. THz radiation is sensitive to the relative permittivity of biological liquids, i.e., their refractive index and absorption, which are directly related to the concentration and type of biological solutes. Recent publications have demonstrated the possibility of studying complex biological systems such as cells and tissues without altering samples and without staining or specific preparation of the sample, in applications such as cancer tissue imaging and diagnosis, cell identification, and electroporation.^[^ ^2^, ^3^, ^4^, ^5^, ^6^, ^7^, ^8^, ^9^, ^10^, ^11^, ^12^, ^13^, ^14^, ^15^ ^]^

Understanding permeabilization processes through plasma cell membranes lies at the core of numerous topics in biology, in particular with regard to pathologies and medical treatments. Moreover, the dynamic aspect of such exchanges is essential, as developing efficient therapies implies the control of timing. To address the topic of exchanges across membranes, biologists rely on either live technique, enabling a continuous recording of specific signals such as electric resistance, or end‐to‐end methods implying analytical tests at different predefined times. These involve preparing multiple experiments to obtain kinetics, associated with the usual required replicates. Classical techniques used to study permeabilization processes are based on imaging and biological techniques, such as electron microscopy, confocal microscopy, or biochemical end‐point quantifications, with either fixed or lysed cells.^[^ ^16^ ^]^ Some real‐time monitoring of plasma membrane integrity exists but displays a number of drawbacks. For example, live videomicroscopy of propidium iodide (PI) penetration is limited by the frequency of acquisition and the cytotoxicity of this DNA intercalant. Transepithelial electrical resistance implies the use of epithelial cells and the formation of a dense monolayer. The development of other techniques capable of demonstrating early leakage processes is therefore essential.

Based on this analysis, we argue that THz spectroscopy could prove itself to be a useful promising and complementary dynamic tool to classical biological end‐point experiments, since it can probe cytosol content without contact or labelling in cell monolayers over a surface of a few square millimeters.^[^ ^10^, ^11^ ^]^ To evaluate this, we have chosen to assess, as an example, the early processes occurring during photodynamic therapy (PDT) in the presence of a photosensitizer, either free or encapsulated in polymeric micelles.

PDT is used clinically to treat various pathologies in dermatology, ophthalmology or oncology.^[^ ^17^ ^]^ It is based on the in situ formation of Reactive Oxygen Species (ROS). These are formed by illumination of a photosensitizer, present at the site to be treated, which, upon excitation, exchanges its energy with the surrounding oxygen molecules. Photosensitizers are mainly polycyclic molecules like porphyrins such as *Pheophorbide a* (Pheo) (structure in Figure S1, Supporting Information). Although clinically used, PDT suffers from limitations such as unspecific biodistribution of the photosensitizer, leading to patient photosensitivity, or difficult light dosimetry. Regarding biodistribution, such a problem being often present for various drugs, various studies have assessed the possible encapsulation of photosensitizers in nanovectors. Indeed, since the early work of Maeda and Matsumura^[^ ^18^, ^19^ ^]^ and the discovery of entrapment of 20–200 nm objects in cancer tumors or inflamed tissues, the so‐called enhanced permeability and retention effect, the development of nanovectors either based on lipids or polymers has burgeoned. Focusing on PDT, different studies have shown that the encapsulation of photosensitizers leads to an improvement of photocytotoxicity, which strongly depends on the nanovector and the cell type used.^[^ ^20^, ^21^, ^22^, ^23^, ^24^, ^25^ ^]^ By working on giant unilamellar vesicles (GUV) of lipids mimicking cell membranes, we also were able to demonstrate that violent processes occurred at the membrane level as soon as illumination was initiated.^[^ ^26^ ^]^ These were very different from one vector to another, either in nature (poly(ethylene oxide)‐*block*‐poly(e‐caprolactone) (PEO‐PCL) versus poly(ethylene oxide)‐*block*‐poly(d,l‐lactide)) or rigidity (original vector vs crosslinked vector). ROS produced by PDT are known to lead to the oxidation of different molecules in the cells, among which are unsaturated lipids. This provokes differences in morphology in the membranes leading to possible exchanges. We were also able to observe this phenomenon by characterizing the oxidation of GUV lipids upon irradiation of Pheo in the presence of different polymeric nanovectors.^[^ ^27^ ^]^

Based on these studies, we decided to examine whether THz spectroscopy could shed light on the differences observed between PDT treatments using either Pheo alone or encapsulated in a polymeric amphiphilic nanovector. The aim of this work is thus to assess THz spectroscopy as an adding‐value technique to supplement usual biological tests and to compare all techniques in a critical manner. The present study therefore focuses on free Pheo and Pheo encapsulated in PEO‐PCL micelles.

## 2 Results and Discussion

In order to grasp the asset or drawback of THz spectroscopy, a thorough evaluation of the information provided by usual biological analyses is essential, keeping in mind that the point is to focus on techniques linked to permeabilization of the cell membrane. It is also important to perform the different analyses on exactly the same systems, either with the usual biological tests or the THz setup. Therefore, the first part of this article will describe the characterization of the state of the membranes and their possible leaking upon PDT process based on already known procedures. Although literature dealing with PDT is wide, these new experiments provide a new insight and enlighten additional interesting data. A second part will detail the THz experiments and discuss the added information thus obtained.

### 2.1 PDT Treatment with Encapsulated Pheophorbide Drastically Alters Cell Integrity

**Figure 1:**
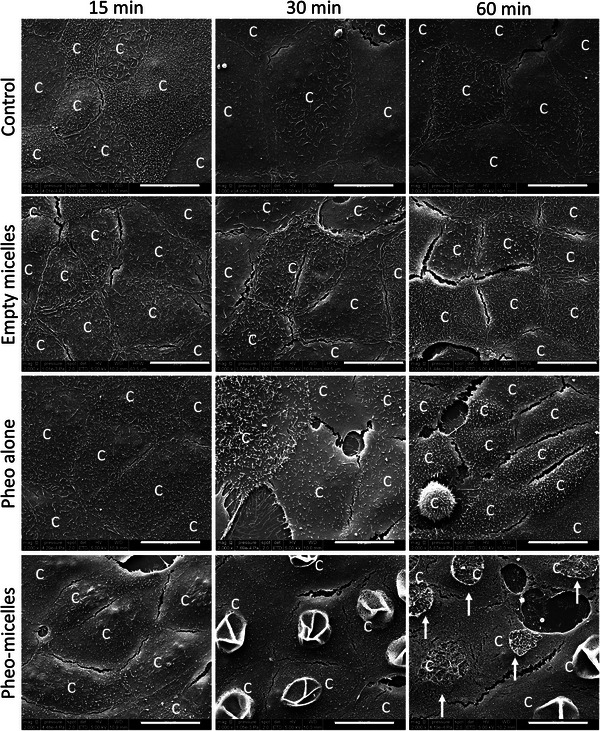
Cell integrity was drastically and rapidly affected by PDT with encapsulated photosensitizer. General aspect of MDCK1 cells was observed by scanning electron microscopy at different short time points after exposure to light irradiation. Pictures are representative of what was observed in the total cell population in two independent experiments. c indicates cells. Arrows indicate unroofed cells. Scale bar = 20 µm.

In a preliminary step, the influence of Pheo concentration on photocytotoxicity was examined in its free form (Pheo alone) or encapsulated in poly(ethylene oxide)‐*block*‐poly(*ε*‐caprolactone) 5000–4000 micelles (Pheo‐micelles) (Figure S1, Supporting Information). As predicted and described in the literature by ourselves and others.^[^ ^28^, ^29^ ^]^ Pheo phototoxicity was significantly increased when encapsulated within micelles compared to its free form. Indeed, in its free form Pheo displays a tendency to form aggregates through *π*–*π* stacking, which decreases its singlet oxygen quantum yield and thus strongly decreases its PDT efficiency.^[^ ^27^ ^]^ A fixed 1.65 µm concentration of Pheo was chosen for subsequent experiments, as such PDT conditions displayed a significant 2.5‐fold factor between free and encapsulated form in terms of viability in MDCK1 (Madin‐Darby canine kidney) cells (Figure S2, Supporting Information). Cell integrity was then observed by scanning electron microscopy (SEM) during the first hour after a sequence of 2 min illumination, 2 min in the dark, and 2 min illumination, corresponding to a total received fluence of 8.2 J cm^−2^ (high‐pass over 400 nm). Cell morphological aspect was undoubtedly different in encapsulated condition Pheo‐micelles from Pheo alone (**Figure** **1**). Indeed, already 30 min after illumination, the plasma membranes appeared destabilized and unroofed cells were visible at 1 h post‐PDT timing. Interestingly, in other conditions (control, empty micelles and Pheo alone), no major modification in cell architecture was seen at these time points in the fixed cells.

### 2.2 PDT with Encapsulated Pheophorbide Induces Rapid and Massive ATP and Ion Fluxes across Plasma Membrane

**Figure 2:**
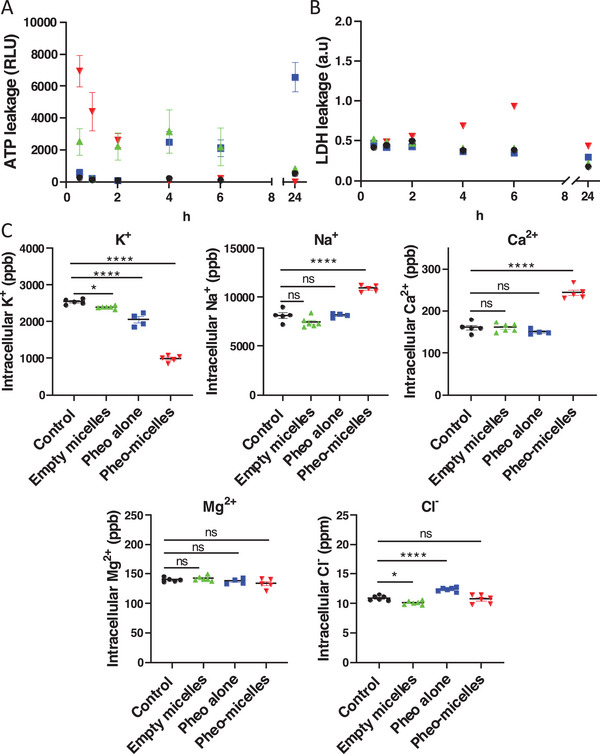
Pheo encapsulation enhances its effects on molecules exchanges across the plasma membrane after PDT treatment. Quantification of molecules of different molecular weights in the extracellular medium at different time points after MDCK1 cells PDT treatment with free or encapsulated photosensitizer. A) ATP release quantified by luminescence. *n* = 6. B) Lactate dehydrogenase enzyme (LDH) activity quantified by absorbance. *n* = 6. C) Ionic movements 30 min after PDT treatment quantified by ICP‐OES and ionic chromatography. *n* = 6. Data are represented as mean ± SEM and analyzed by one‐way ANOVA followed by Dunnett's multiple comparisons test, which compare all conditions with the control condition. ● = control, 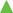 = empty micelles,  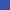 = Pheo alone,  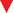 = Pheo‐micelles.

In order to go further into the characterization of the phenomena occurring after illumination at plasma membrane scale, and to shed light on the size of the possible plasma membrane defects, exchanges of several molecular species were quantified at different time points after PDT treatment. For this purpose, extracellular adenosine triphosphate (ATP) (507 Da) and extracellular lactate dehydrogenase (LDH) (140 kDa) were quantified by absorbance over 24 h using end‐point luminescence and enzyme activity respectively (**Figure** **2**). A rapid and massive ATP leakage was quantified for encapsulated Pheo‐micelles from the first time point the analysis took place (30 min after light irradiation) and then decreased until it was no longer detectable, 4 h after treatment (Figure 2A). On the contrary, for Pheo alone, extracellular ATP increased regularly over time after light irradiation. It is interesting to note that the kinetics were clearly different for free and encapsulated Pheo. It means that cell mechanisms involved in ATP release in the extracellular medium are different, as can be observed in plasma membrane changes occurring during distinct cell death pathways.^[^ ^30^ ^]^ While PDT treatment is known to induce immunogenic cell death,^[^ ^31^ ^]^ it appears that encapsulating the photosensitizer could significantly improve the release of ATP, which is one of the main hallmarks of immunogenic cell death. Worthy of note is that empty micelles induced a constant leakage of ATP. We previously showed that empty amphiphilic PEO‐PCL micelles were able to destabilize liposome membranes as well as be internalized by cells.^[^ ^27^ ^]^ This observation could possibly be related to what is known about membrane integration of amphiphilic pluronic copolymers.^[^ ^32^ ^]^ With regard to LDH release, which is classically used as a cytotoxicity assay to monitor the level of plasma membrane damage in a cell population, a difference in cell behavior when exposed to free or encapsulated Pheo was confirmed (Figure 2B). LDH was increasingly found in the extracellular medium when cells were exposed to encapsulated Pheo while remaining undetectable in other conditions. These quantitative data are consistent with the SEM images showing significant early cell damage when the protocol was performed in the presence of encapsulated Pheophorbide. The same experiments carried out without light irradiation indicated that no leakage of ATP or LDH was detectable in this control condition, underlying the major role of the Pheophorbide photosensitizer in light activation (Figure S3, Supporting Information).

Intracellular ions were quantified by inductively coupled plasma optical emission spectrometry (ICP‐OES) and ionic chromatography at a fixed time point of 30 min after PDT treatment (Figure 2C). These techniques were chosen because they enabled the analysis of all cations and anions simultaneously, without focusing on one specifically. Light irradiation on its own did not modify the intracellular content in the five ions under consideration, namely Cl^−^, Ca^2+^, Na^+^, K^+^, and Mg^2+^ (Figure S4, Supporting Information). Notably, empty micelles led to a slight but significant efflux of K^+^ and Cl^−^ ions. Together with the ATP leakage, these results shed light on the effective interaction of polymer on its own with plasma membrane. Previous published studies indicated that ion fluxes across plasma membrane were dependent on photosensitizer concentration in cells or durations of light irradiation, in other words they were a function of ROS production and plasma membrane damages. In our experimental setup, a massive leakage of K^+^ ion was quantified 30 min after PDT, corresponding to a twofold decrease in encapsulated Pheo condition compared to its free form. Rapid and massive leakage of K^+^ was previously reported in the literature^[^ ^33^ ^]^ and thought to be independent of Na^+^/K^+^‐ATPase, since it was not associated with a concomitant Na^+^ influx.^[^ ^34^ ^]^ In our experimental setup, Na^+^ influx was indeed insignificant in the free Pheo condition but proved to be the opposite in the encapsulated condition, meaning that nonimplication of Na^+^/K^+^‐ATPase should not be taken for granted in the case of encapsulated photosensitizer. Ca^2+^ intracellular content increased drastically and solely for the encapsulated Pheo condition. This observation is not fully in accordance with previous studies reporting a rapid increase in intracellular Ca^2+^ content after PDT with different free photosensitizers such as photofrin,^[^ ^35^, ^36^ ^]^ 2‐butylamino‐2‐demethoxy‐hypocrellin A,^[^ ^37^ ^]^ or hematoporphyrin derivatives.^[^ ^38^ ^]^ No modification in intracellular Mg^2+^ content was observed 30 min after light irradiation, which is consistent with the fact that most intracellular magnesium exists in a form bound to other biomolecules.^[^ ^39^ ^]^ Finally, Cl^−^ ions significantly entered cells following light irradiation of the free pheo, whereas this was not the case for the encapsulated pheo. At this point no explanation can be advanced, especially because no information was found in the literature concerning Cl^−^ exchanges after PDT treatment. Our results first indicated ionic movements across plasma membrane after PDT treatment and secondly a clear difference in ion fluxes in response to free or encapsulated PDT, which will require further exploration to understand cellular underlying mechanisms.

### 2.3 Loss of Plasma Membrane Integrity Depends on Pheophorbide Concentration and Encapsulation

**Figure 3:**
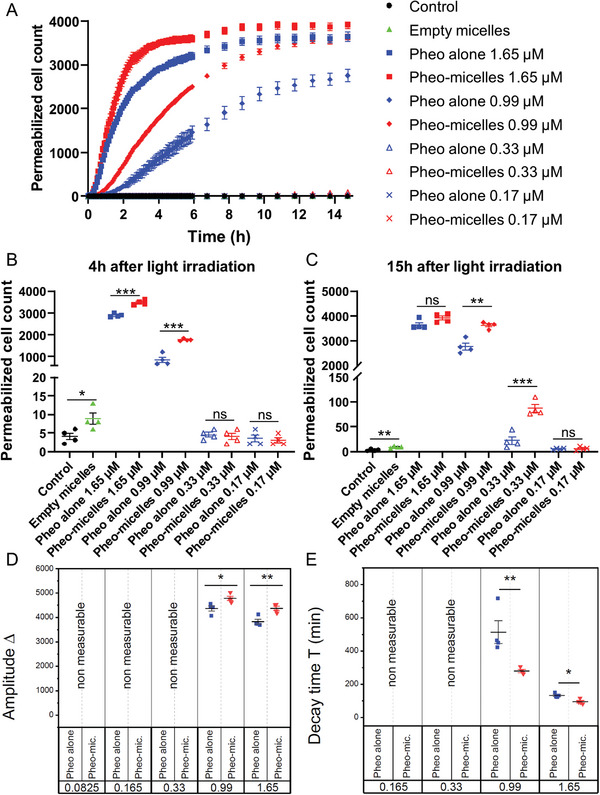
Pheo encapsulation accentuated its effects on loss of plasma membrane integrity. A) Real‐time monitoring of plasma membrane integrity through propidium iodide penetration, quantified by fluorescence videomicroscopy over time after PDT treatment with increasing concentrations of free or encapsulated photosensitizer. B) Focus on 4 h time point after light irradiation. C) Focus on 15 h time point after light irradiation. D) Amplitude parameter Δ extracted from propidium iodide penetration observed by videomicroscopy. E) Decay time *T* for fluorescence extracted from propidium iodide penetration observed by videomicroscopy. Data are represented as mean ± standard error of the mean and analyzed using unpaired *t*‐test. *n* = 4. 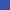 = Pheo alone, 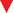 = Pheo‐micelles.

While these previous biological experiments were based on end‐point measurements, we made use of videomicroscopy to monitor plasma membrane integrity over time. The permeability of plasma membrane was thus examined thanks to PI penetration with decreasing concentration of free or encapsulated Pheo (**Figure** **3**A). For the first 4 h, pictures were acquired every 5 min and then every hour. Because of the number of conditions and replicates to be acquired in the same experimental batch, we were not technically able to acquire pictures at a higher frequency. The earliest detection of PI penetration, meaning loss of plasma membrane integrity, was recorded 20 min after light irradiation of the highest free or encapsulated Pheo concentration (1.65 µm). The rapid onset of membrane effects following PDT treatment has already been observed by videomicroscopy in the case of another free photosensitizer, namely cationic phthalocyanine.^[^ ^40^ ^]^ While at 4 h after light irradiation, no significant difference was observed in plasma membrane integrity between free and encapsulated Pheo at lowest concentrations 0.33 and 0.17 µm (Figure 3B), some differences were detectable at 15 h after light irradiation, at least in the case of the 0.33 µm concentration (Figure 3C). Interestingly, whatever the tested concentration was, the encapsulated condition always led to higher PI penetration than free Pheo, meaning more plasma membrane defects. The lower the photosensitizer concentration, the more important the benefit of encapsulation. Therefore, 15 h after light irradiation (Figure 3C), Pheo encapsulation increased cell permeability by a factor of 1.1, 1.3, and 4.0 for 1.65, 0.99, and 0.33 µm conditions respectively.

Monitoring membrane integrity through propidium iodide penetration by videomicroscopy at concentration 0.99 µm allowed extracting the normalized amplitude variations Δ (Figure 3D) and the characteristic time decay *T* (Figure 3E) (Figure S7, Supporting Information). The amplitude *Δ* describes the relative variation of the signal before illumination and after a long delay time and is representative of the number of molecules exchanged through the membrane. These quantitative parameters confirmed that Pheo encapsulation increased plasma membrane permeability after PDT treatment. For concentrations below 0.99 µm, these parameters could not be determined due to noise level.

### 2.4 Permeabilization Kinetics Examined by THz Spectroscopy

**Figure 4:**
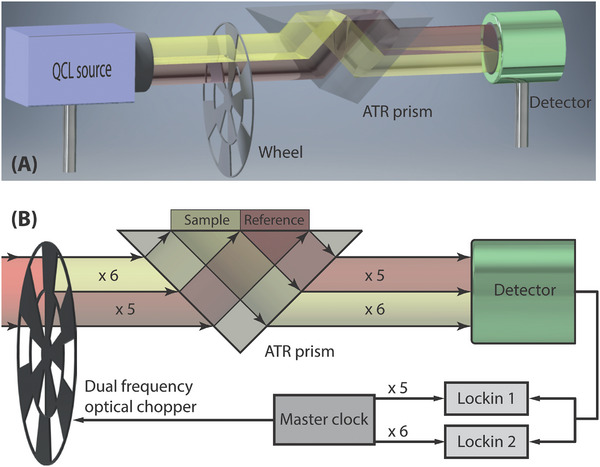
Dual‐modulation differential ATR sensor setup. A) 3D view. B) Detailed view. The THz beam from the QCL source is chopped at two different frequencies x5 and x6 by the two rows of chopper slots. Half of the beam is modulated at x6 and is reflected by the sample, while the other is modulated at x5 and used as a reference. The two halves are recombined and sent to the same detector. Two lock‐in amplifiers are used to detect the sample and reference signals. The chopper controller provides the modulation signals for the optical chopper and lock‐in amplifiers.

The THz setup (**Figure** **4**) was based on a quantum cascade laser (QCL) coupled to an attenuated total reflectance system (ATR) made of a high‐resistivity (HR) silicon prism on which the cell culture was implemented in a temperature‐controlled atmosphere. The key point of the setup is a mechanical chopper which simultaneously modulates the THz beam at two different frequencies. For each measurement, MDCK1 cells were grown on an HR silicon plate and placed on top of the ATR prism. Our THz sensor is a differential ATR setup with a capacity to detect a relative reflectivity variation of 10^−4^, corresponding to a micromolar level of detection of molecules in solution.

**Figure 5:**
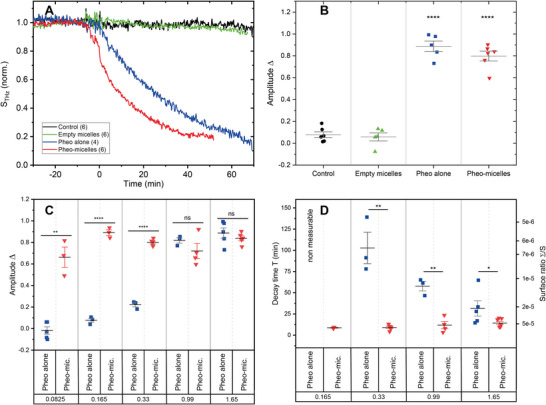
Dynamics of plasma membrane permeabilization following free or encapsulated PDT, analyzed by THz‐ATR. A) Summary of illuminated conditions. Mean values (number of replicates is indicated in brackets) are represented. Concentration of Pheophorbide is 1.65 µm. B) Amplitude parameter *Δ* for all conditions. C) Amplitude parameter *Δ* for illuminated condition in presence of Pheo alone and Pheo‐micelles, for different concentrations (in µm). D) Delay time *T* for illuminated condition in presence of Pheo alone and encapsulated Pheo in micelles, for different concentrations (in µm). The right‐hand axis gives the relative number of defects per membrane surface Σ/*S*. Values at 0.0825 and 0.165 µm are not accessible for Pheo alone since the amplitude of the signals is close to 0. ■: pheo alone condition; ▼: pheo encapsulated in micelles condition; ▲: empty micelles condition; and ●: control condition.

THz attenuated total reflection (THz‐ATR) makes use of the evanescent wave at the back of a prism under total internal reflection,^[^ ^41^, ^42^ ^]^ which is coupled to the sample under study (Figure 4 and Figure S5, Supporting Information). Providing that the thickness of the cell layer matches the penetration depth of the evanescent wave, the reflected THz wave is modified by the THz relative permittivity value and repartition in the cell layer in contact with the top of the prism. In our experiments, the cell layer thickness is 10 ± 2 µm, which matches very well with a penetration depth of 8 µm at 2.5 THz. The THz‐ATR sensor then allows real time and continuous measurement of the change in the cell cytosol concentration with a time resolution of a few seconds. Such dynamics are presented in **Figure** **5** on MDCK1 cells under control (illumination alone), Pheo and Pheo‐micelles conditions with illumination (all experiments with and without illumination can be found in Figure S6, Supporting Information). Results with a concentration of free or encapsulated Pheophorbide solutions set at 1.65 µm are detailed in Figure 5A. The control experiments show no modification of the THz‐ATR signal, while Pheo and Pheo‐micelles experiments show dynamics with a large amplitude. Typical dynamics times are in tens of minutes. More precisely, Pheo‐micelles conditions demonstrate a much faster response than Pheo alone.

From the THz‐ATR dynamics and similarly to the treatment of videomicroscopy data, we extracted the normalized amplitude variations *Δ* and the characteristic time decay *T* (Figure S7, Supporting Information). As expected from the THz‐ATR signals (Figure 5A), the amplitude *Δ* is very close to 0 for all measurements but the ones with free and encapsulated Pheo under illumination conditions at concentration of 1.65 µm (Figure 5B). For these two conditions, the amplitude for both is above 80%, meaning a very large modification of the cytosol within less than an hour after illumination. The effect of concentration was also investigated. The amplitude *Δ* of free (Pheo) and encapsulated (Pheo‐micelles) Pheo are compared in Figure 5C for concentrations ranging from 0.0825 to 1.65 µm. No significant effect of encapsulation is observed at the highest concentrations of 0.99 and 1.65 µm. On the contrary, for concentrations below 0.33 µm, the effect of encapsulation is very significant, even at concentration as low as 0.0825 µm where Pheo alone has no observable effect on the THz‐ATR signal, while Pheo‐micelles still strongly modify cytosol content. The decay times for encapsulated Pheo (Figure 5D) were always smaller than without encapsulation, meaning that encapsulation quickened the cellular alterations. This effect was even more pronounced at lower concentrations. Interestingly, the THz spectroscopy approach allowed visualization and quantification of plasma membrane alterations for very low concentrations of photosensitizer, highlighting the added value of encapsulation, whereas the classical biology technique of propidium iodide penetration monitoring was not sensitive enough to obtain such information. Indeed, by performing the same type of quantitative analysis of *Δ* (Figure 5C) and T (Figure 5D) we obtained slightly different results from the experimental data obtained by videomicroscopy (Figure 3D,E). It is clear that videomicroscopy is less sensitive than THz as it was not able to detect significant variations in both *Δ* and *T* for low Pheophorbide concentrations, i.e., below 0.99 µm. The other extremely interesting point is the time scale for visualizing/quantifying membrane effects of PDT treatment: while THz revealed these effects immediately after illumination, it was necessary to wait several minutes or even hours for the lowest Pheo concentrations to detect a significant variation in membrane permeability by videomicroscopy. THz spectroscopy is thus more sensitive than classical biological approaches. On the other hand, it should be stressed that while the biological approaches used in this work can provide information on the nature of the cellular and molecular events occurring after PDT (type and kinetics of cell death, redistribution of ions in different compartments, etc.), THz spectroscopy does not provide this type of information.

Importantly, the time T can be related to the amount of membrane defects created during illumination, by an effusion model derived from Fick's second law.^[^ ^43^ ^]^ In contrast to our previous study using chemical permeabilization with saponin^[^ ^10^ ^]^ where membrane defects were created continuously, we consider that the defects produced during PDT occurred only during illumination. Therefore, the inverse of the time T is proportional to the total area of the membrane defects according to the following relationship

(1) $$\frac{1}{T}=\frac{\Sigma }{S}\;\frac{D_{c}}{L\;\delta }$$

where Σ is the total defect surface, *S* is the membrane surface, *D*_c_ is the diffusion constant of the leaking molecules from the cytosol, *L* is the cell height, and *δ* is the membrane thickness. The more effective the photosensitizer is, the larger the defects surface created, the faster the dynamics and therefore the smaller *T* is. With *D*_c_ = 1µm^2^ s^−1^, *L* = 10 µm, and *δ* = 4 nm,^[^ ^10^ ^]^ one can calculate for each THz dynamics the relative number of defects per membrane surface Σ/*S*, as shown in Figure 5D. For instance, an average time *T* = 30 min is measured for THz PDT dynamics for Pheo alone at 1.65 µm, which corresponds to a ratio Σ/*S* ≈ 2 ×10^−5^. The direct comparison between the experiments is even more accurate. At 1.65 µm, the relative defect surface with Pheo‐micelles is 2.1 times higher than with Pheo alone. It is 11 times higher at 0.33 µm, which clearly demonstrates and also quantifies the large increase of defect production when using micelles. Thus, the parameters extracted from the THz signal allowed the reliable quantification of plasma membrane defects generated by a specific stress, the oxidative stress induced by PDT in our case.

Interestingly, THz‐ATR seems to reveal that the membrane permeabilization process by Pheophorbide is saturable. The maximum of changes in the composition of cytosol content is reached for the lowest Pheo‐micelle concentration (0.0825 µm), which will be achieved by Pheo alone at the highest concentration of 1.65 µm. These results raise an interesting question: would this signal amplitude or reaction time decay represent the signature of a photosensitizer? THz‐ATR would be a powerful technique to study and compare the efficacy of photosensitizers for photodynamic application. Future investigations could examine this, but this lies beyond the scope of the present work.

This work puts forward two types of information, the first linked to PDT itself and the other to the strength of THz spectroscopy. Regarding the knowledge generated on PDT in this study, several points can be noted: 1) major membrane events appear upon illumination and induce massive cell responses within minutes; this kinetic and rapid aspect is to be compared with studies that have well characterized the different types of PDT‐induced cell death, whether conventional or nonconventional;^[^ ^44^ ^]^ 2) even if encapsulation of photosensitizer is widely known to improve PDT therapeutic efficiency both in vitro and in vivo, we were able for the first time to quantify the plasma membrane defect areas, depending on the formulation of the photosensitizer (free or encapsulated), exclusively thanks to THz‐ATR spectroscopy. Regarding THz spectroscopy, only a couple of studies have previously examined the dynamics of cell membrane integrity. Grognot et al. demonstrated the uses of THz‐ATR in the sensing of cell membrane permeabilization after addition of saponin using THz time‐domain‐spectroscopy (THz‐TDS), which enabled the evolution of the THz signal after excitation to be shown, but with a low signal resolution, thus preventing the precise analysis of the dynamics.^[^ ^45^ ^]^ Very similarly, Zou et al. also used THz‐TDS to study oxidative stress in MCF10 breast epithelial cells.^[^ ^8^ ^]^ They followed the amplitude of the THz pulse in time‐domain and correlated its evolution to the cell death rate obtained from flow cytometry using propidium iodide and annexin‐V over a 2 h period. Even if a high concentration of 10 mm H_2_O_2_ was used, only small evolutions were observed, which also prevented the precise analysis of the dynamics. Finally, more precision was achieved by Zheng and Gallot^[^ ^10^ ^]^ and Azan et al.^[^ ^11^ ^]^ by averaging numerous experiments, thus enabling the extraction of relevant quantitative experimental parameters from their matching with biophysical models. Here, we achieved THz measurements with much better precision and stability thanks to our new experimental setup at 2.5 THz. It allows the recording of precise single measurement data and the extraction of the relevant parameters of each measurement. Furthermore, in our case, we were able to highlight very early membrane integrity loss which was not observable in any other classical biological techniques assessed so far in this study. More specific technics such as electrophysiology, voltage‐sensitive membrane probes, and calcium or pH fluorescence probes could also be helpful in deciphering finely cell mechanisms involved. In particular, when compared to fluorescence measurements, THz spectroscopy provides a direct, nondestructive, and quantitative measurement without sample preparation or labelling, while fluorescence microscopy requires fluorescent molecular probes that are potentially (photo)toxic to cells, whose quantum yield depends on the microenvironment, and may suffer from photobleaching. Thus, quantitative measurements are difficult to obtain. In contrast, THz sensing allows long term measurements. Nevertheless, the transverse resolution of fluorescent imaging is much better than the THz resolution, which is limited to a fraction of a millimeter by diffraction. Another limitation of THz‐ATR is the lack of sharp spectroscopic features compared to the infrared range. Nowadays, THz‐ATR measurements are only performed at a fixed frequency, but thanks to technological developments, broadband terahertz time‐domain spectroscopy systems are now commercially available and can be used for THz‐ATR, so that much more detail about the size and chemical nature of the molecules in the cytosol will be obtained using THz‐TDS‐ATR. THz spectroscopy is, therefore, a very efficient, promising and complementary technique to the established biological tests in the study of short‐term modifications of plasma membrane permeability after chemical^[^ ^8^, ^10^ ^]^ or electrical^[^ ^11^ ^]^ treatments. This technique could also be applied more generally to monitor the state of cells (cell differentiation, cancer or healthy cells) or to study the adaptation of cells to new environments (cell structure and integrity, hydration imbalance, glycogen storage, ion channel related diseases, etc.) leading to a modulation in the cytosolic content.

## 3 Conclusion

This work has proved that THz‐ATR is a powerful, promising and complementary technique to other biological approaches to monitor changes in cell permeability upon a photochemical stress, especially thanks to its high sensitivity and capacities to enable the early detection of membrane permeabilization processes in real‐time on living cells. The THz‐ATR has also proven to be a very powerful tool for studying the effectiveness of copolymer‐based nanovectors as an agent for drug delivery, including photosensitizers. We show in our work that, thanks to the tremendous recent development of THz sources, this spectroscopy now has the possibility to be applied to biology in an innovative and efficient way. The THz setup enabled to highlight very early membrane alteration, an achievement not currently possible with any other biological technique assessed so far. In particular, it provides in a single measurement the relative number of defects per membrane surface created after PDT and allows direct quantification of the effects of micelle vectorization on the increase of defects. These very original results offer a wide field of applications for THz‐ATR in the field of nanovectors for drug delivery, allowing to better determine their interaction with cell membranes. The development of a repertoire of THz‐ specific signatures of different biological molecules would help to further identify the biological processes involved at cell scale after stimulus. Thus, it appears plausible to implement this biophysical technology in several labs to address more largely biological questions, paving the way for new biological/biomedical investigations dealing with cell death, cell growth, cell communication, and responses to various stresses or therapeutic strategies.

## 4 Experimental Section

### Chemicals

PEO‐PCL 5000–4000 was purchased from Polymer Source (Polymer Source, Inc., Dorval, Canada). Pheophorbide‐a (Pheo) was purchased from Wako (Japan). Acetone and phosphate buffer saline (PBS) were purchased from Sigma‐Aldrich (Merck, Darmstadt, Germany). The water used was ultrapure water at 18 MΩ cm resistivity obtained from an ELGA Purelab Flex system (ELGA LabWater, High Wycombe, UK).

### Preparation of Block‐Copolymer Micelles

PEO‐PCL block‐copolymer micelles were prepared and characterized as previously described.^[^ ^46^ ^]^ Briefly, 20 mg of PEO‐PCL copolymer were dispersed in 400 µL of acetone. This solution was slowly added to 5 mL of water whilst stirring over a 5 min period. The solution was left standing in the open air to evaporate the acetone for 48 h. For all samples loaded with Pheo, a stock solution of the photosensitizer in acetone was used to dissolve the polymer prior to its addition to the water solution. The molar ratio between the photosensitizer and the polymer was kept constant at 1/30 mol/mol to ensure its complete encapsulation. The hydrodynamic sizes of the carriers were determined by dynamic light scattering (DLS) on a Malvern Zetasizer instrument, using the General Purpose, non‐negative least square routine for data processing. Characteristics of empty micelles and Pheo‐micelles are reported in **Table** **1** and Figure S8 in the Supporting Information. The shown averages (standard error of mean) were obtained from 15 different experiments on randomly chosen dates.

**Table 1:** DLS characterization of empty micelles and Pheo‐micelles. Data are represented as mean (standard error of mean), *n* = 15 independent experiments

Sample	Intensity‐mean DLS size [nm]	Number‐mean DLS size	Polydispersity index
Empty micelles	32.3 (2.0)	18.7 (1.1)	0.36 (0.06)
Pheo‐micelles	37.1 (2.6)	14.3 (0.5)	0.33 (0.07)

### Cell Culture

MDCK1 cells, reported to form a monolayer with characteristics of tight epithelia of the renal collecting duct, were recently purchased from European collection of authenticated cell cultures (Sigma #62106, St Quentin Fallavier, France). Cells were grown in Dulbecco's Modified Eagles Medium (ThermoFisher # 61965, Carlsbad, CA, USA) containing GlutaMAX, 4.5 g L^−1^ glucose, supplemented with 10% v/v heat‐inactivated fetal calf serum, 100 U mL^−1^ penicillin and 100 µg mL^−1^ streptomycin. Throughout the experiments, cells were tested negative for mycoplasma (MycoAlert mycoplasma detection kit, Ozyme #LT07‐318, St Cyr l’École, France). Cell cultures were maintained in a humidified atmosphere at 37 °C containing 5% CO_2_ and culture media were changed three times a week.

### Photodynamic Therapy of Cell Monolayers

Cells were allowed to grow for 48 h before treatment to reach 95–100% confluency. The cell seeding density was set to 31 000 cells cm^−1^. Cells were incubated for 30 min at 37 °C with nothing (control condition), free 1.65 µm photosensitizer *Pheophorbide a* (Pheo alone), empty micelles, 1.65 µm pheo‐micelles before being submitted to light irradiation as previously described.^[^ ^27^ ^]^ A spotlight protected by a glass slide (band pass filter *λ* > 400 nm) was placed 4 cm above the samples. Cells were light‐irradiated for a total of 4 min (2 min light on, 2 min light off and then 2 min light on in order to avoid heating). Each was exposed to a total of 8.2 J cm^−2^. After light irradiation, the cells were placed back in the incubator until analysis. A positive control of chemically induced cell permeabilization was added, using Saponin (Merck #47036, Darmstadt, Germany) at 75 µg mL^−1^ in PBS.

### SEM

For surface analyses, MDCK1 cells were grown in monolayer on 12 mm diameter round glass coverslips for 48 h in 24‐well plates. Since cells are not supposed to grow on glass, these coverslips were coated with gelatine (Fisher Scientific) chosen because of its high biocompatibility with cells.^[^ ^23^ ^]^ On the day of treatment, after 30 min incubation in respective conditions (**Table** **2**), cells were light irradiated as described above. After 5, 15, 30, and 60 min of postlight irradiation, the cell culture medium was thoroughly aspirated and cells were fixed in 2% glutaraldehyde in a 0.1 m Sorensen phosphate buffer (pH 7.4) at 4 °C until further being processed for scanning electron microscopy. Observations were carried out with a Quanta 250 FEG FEI scanning electron microscope.

**Table 2:** Summary of the area and frequencies covered by the complementary biological and spectroscopic analyses used in our experiments

	Cell area analyzed	Frequency
Scanning electron microscopy (SEM)	113 mm^2^	Endpoint
ATP/LDH leakage	32 mm^2^	Endpoint
Ion fluxes (ICP‐OES/ion chromatography)	960 mm^2^	Endpoint
Propidium iodide penetration (videomicroscopy)	2.1 mm^2^	Every 5 min
THz‐ATR spectroscopy	20 mm^2^	Real‐time

### ATP Leakage Quantification

Extracellular ATP was quantified in the cell culture medium at different time points (30 min and 1, 2, 4, 6, and 24 h) after treatment. For that purpose, a CellTiter‐Glo Luminescent cell viability assay (Promega #G7570) was adapted in order to quantify cytoplasmic ATP released into the extracellular medium. Briefly, 45 µL of supernatant were mixed with 45 µL of CellTiter‐Glo Reagent in white 96‐well plates, incubated and protected from light at room temperature for 5 min before luminescence was read on a Synergy H1 plate reader (Biotek, Winooski, VT, USA).

### LDH Leakage Quantification

LDH activity was quantified in cell culture medium at different time points (30 min and 1, 2, 4, 6, and 24 h) after treatment. It is an accurate way to determine plasma membrane integrity as a function of the amount of cytoplasmic LDH released into the medium. For that purpose, LDH Assay (Sigma, #TOX7, St Quentin Fallavier, France) was performed according to the manufacturer's protocol. Note that to perform a reliable quantification, fetal calf serum added to cell culture medium has to be heat‐inactivated (30 min at 56 °C), as it contains LDH residual activity.^[^ ^47^ ^]^ Briefly, 45 µL of supernatant were mixed with 90 µL of LDH mix (Substrate Solution: Dye Solution: Cofactor Preparation) in 96‐well plates, incubated protected from light at room temperature for 20 min before absorbance was read at 490 nm on a Synergy H1 plate reader (Biotek, Winooski, VT, USA).

### Ion Flux Analysis

300 000 MDCK1 were plated in six‐well plates 48 h before treatment. Cells were incubated for 30 min at 37 °C with empty micelles, Pheo alone, or Pheo‐micelles in Hank's Balanced Salt Solution (HBSS) (Fisher 14025050) in order to be in similar conditions as for THz experiments. After incubation, cells were light irradiated as described before. Cells were then placed in the incubator at 37 °C. After 30 min, the treatment medium was thoroughly aspirated and cells were extracted using a cell scraper and left for 30 min at room temperature in 10 mL of ultrapure water. These 10 mL of water containing intracellular content were filtered through a 0.22 µm filter. 600 µL were dedicated to intracellular total anion quantification using high‐performance ion chromatography (Dionex ICS‐2000) and the rest of the liquid was supplemented with 200 µL of concentrated HNO_3_ (65%) to obtain intracellular total cation measurements, performed using ICP‐OES with an HORIBA Jobin Yvon Expert.

### Propidium Iodide Penetration

Plasma membrane defects were visualized using propidium iodide (Merck #P4170, Darmstadt, Germany) in real time thanks to videomicroscopy. Propidium iodide is a nonpermeant fluorescent DNA intercalant, meaning penetration occurs only inside cells presenting loss of plasma membrane integrity. Briefly, cells grown in 96‐well plates were treated by PDT as described in Table 2 and were incubated in 1 µm propidium iodide immediately after light irradiation. Plates were then placed immediately within an IncuCyte S3 live cell imaging system (Sartorius). Pictures in red fluorescence were acquired every 5 min during the first 4 h and then every hour, with quantifications performed using software linked to the video microscope. Film footage depicting propidium iodide penetration into cells after PDT treatment is available in Movie S1 in the Supporting Information.

### THz Spectroscopy

The setup is based on a THz QCL source (TeraCascade 1000, Lytid) emitting 3 mW in continuous radiation at 2.5 THz. The divergent output beam is collimated by an *f* = 50 mm, *D* = 50 mm, high‐resistivity silicon (HR‐Si) lens. The beam is then reduced to a 4 mm full width at half maximum collimated beam using an afocal optics consisting of an off‐axis parabolic mirror (*f* = 150 mm) and an HR‐Si lens (*f* = 25 mm) with a reduction ratio of 6. All HR‐Si lenses are antireflection‐treated at 2.5 THz to reduce reflection loss. The beam is then directed to the ATR sensor described in Figure 4. The beam passes through a dual frequency optical chopper and is simultaneously chopped at two different frequencies. The upper half of the beam is chopped by six outer slots, with the lower half chopped by five inner slots. Thus, the upper and lower halves of the beam are modulated, relatively to a master frequency set at about 65 Hz, at frequencies of ×6 (390 Hz) and ×5 (325 Hz), respectively. Both halves then undergo an ATR on the HR‐Si prism (50 × 50 mm input face, angle of 42°) in two separate positions, where the sample and reference liquids can be placed. After exiting the prism, both parts of the parallel beam are focused on a pyroelectric detector with a surface area of 4 mm^2^ (TeraPyro, Lytid, 390 V W^−1^ sensitivity) by an HR‐Si lens (*f* = 25 mm). The pyroelectric signal, resulting from the superposition of the two modulations, is demodulated by two independent lock‐in amplifiers driven by each of the two frequencies provided by the chopper controller. One obtains the signals *S*_5_ (×5) and *S*_6_ (×6) for both modulations. The whole setup is placed in a sealed box with controlled humidity (below 2% relative humidity) and temperature (21 °C). The ATR prism is also precisely thermalized at 37 ± 0.01 °C using two Peltier thermoelectric coolers and a temperature controller (Thorlabs TED200C). In order to cancel the residual fluctuations, the THz signal *S*_THz_ used for the measurements is calculated as *S*_THz_ = *S*_5_ /*S*_6_. The THz‐ATR sensor is thus characterized by an excellent signal‐to‐noise ratio and stability in the long term (< 10^−3^). Since the ATR prism cannot easily be removed, the MDCK1 cells are grown on a separate 3 mm thick HR‐Si plate which is put on top of the ATR prism. A small drop of *α*‐pinene (Sigma‐Aldrich, P45702) is used as an index‐matching layer ensuring the optical continuity between the prism and the cell plate. The cell layer is carefully monitored to ensure that it does not peel off during the experiments.

### Comparison of Different Analytical Techniques

Table 2 summarizes the area and frequencies covered by the complementary biological and spectroscopic analyses used in the experiments.

### Statistical Analyses

Data analysis was performed using GraphPad Prism 8 Software (La Jolla, CA, USA) and Origin 2019 (OriginLab) for THz data, with independent biological replicate data individually plotted and expressed as mean ± SEM (standard error of mean). Data were statistically analyzed using one‐way analysis of variance (ANOVA) followed by Dunnett's post‐test to compare every condition to the control one. Statistics are expressed relative to the control condition. ns: nonsignificant, * *p* < 0.05, ** *p* < 0.01, *** *p* < 0.001, and **** *p* < 0.0001.

### Ethics Approval

No ethical issue was raised during this study because cells used were commercially available.

## Conflict of Interest

The authors declare no conflict of interest.

## Author Contributions

A.‐F.M., P.V., L.G., and G.G. designed research. X.Z., B.L., A.‐F.M., P.V., R.B., I.F., L.G., and G.G. performed research. X.Z., B.L., and G.G. performed terahertz experiments. R.B., I.F., and L.G. performed the classical biological experiments. X.Z., A.‐F.M., P.V., L.G., and G.G. analyzed data. A.‐F.M., P.V., L.G., and G.G. wrote the paper.

## Supporting information

Supporting Information

Supplemental Movie 1

## Data Availability

The authors declare that the main data supporting the findings of this study are available within the article and its Supporting Information. Extra data, including details of statistical analyses, are available from the corresponding author upon reasonable request.
