# Supplementary material for: Terahertz Spectroscopy Sheds Light on Real‐Time Exchange Kinetics Occurring through Plasma Membrane during Photodynamic Therapy Treatment
Source: Adv Sci (Weinh). 2023 Apr 25;10(18):2300589. doi: 10.1002/advs.202300589 (PMC10288265; doi:10.1002/advs.202300589)
Supplement: Supplementary file 1 — Supporting Information [file ADVS-10-2300589-s002.pdf]

ADVANCED SCIENCE
Open Access

Supporting Information

for *Adv. Sci.*, DOI 10.1002/advs.202300589

Terahertz Spectroscopy Sheds Light on Real-Time Exchange Kinetics Occurring through Plasma Membrane during Photodynamic Therapy Treatment

Xiujun Zheng, Blandine Lordon, Anne-Françoise Mingotaud, Patricia Vicendo, Rachel Brival, Isabelle Fourquaux, Laure Gibot and Guilhem Gallot**

Supporting Information

Terahertz spectroscopy sheds light on real-time exchange kinetics occurring through plasma membrane during photodynamic therapy treatment

Xiujun Zheng, Blandine Lordon, Anne-Françoise Mingotaud, Patricia Vicendo, Rachel Brival, Isabelle Fourquaux, and Laure Gibot, Guilhem Gallot**

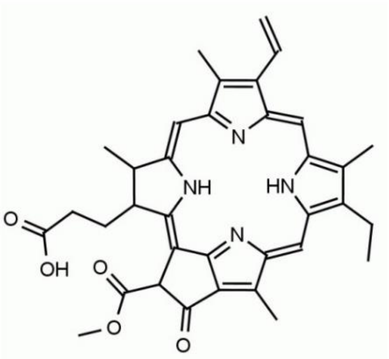

Pheophorbide a

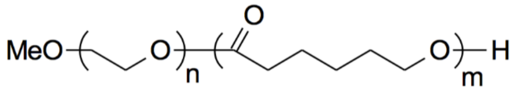

PEO5000-PCL4000

Poly(ethylene oxide) - *block*- poly(ε - caprolactone)

Figure S1. Structures of *Pheophorbide a* and poly(ethylene oxide)-*block*-poly(e-caprolactone) 5000-4000. 5000-4000 indicates the molar mass of each block, PEO and PCL respectively. This corresponds to mean values of 113.6 for n and 35.1 for m.

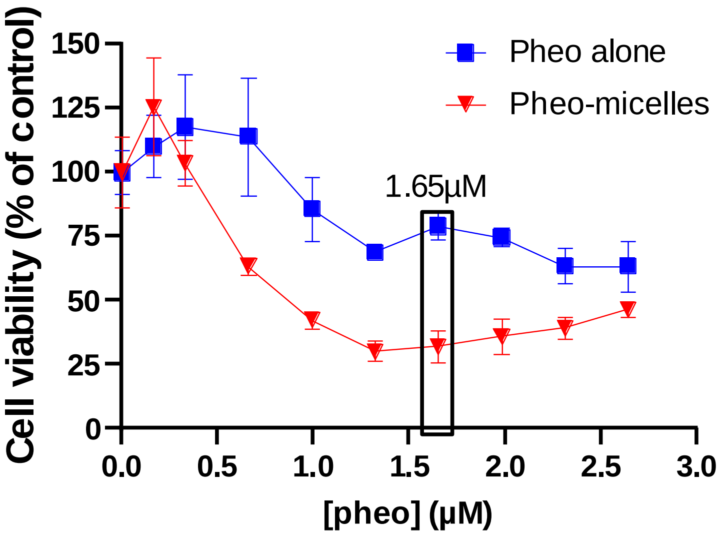

Figure S2. Encapsulation of Pheophorbide drastically alters cell viability 24h after PDT, assessed by Prestoblue assay. Data are represented as mean ± SEM. n=3.

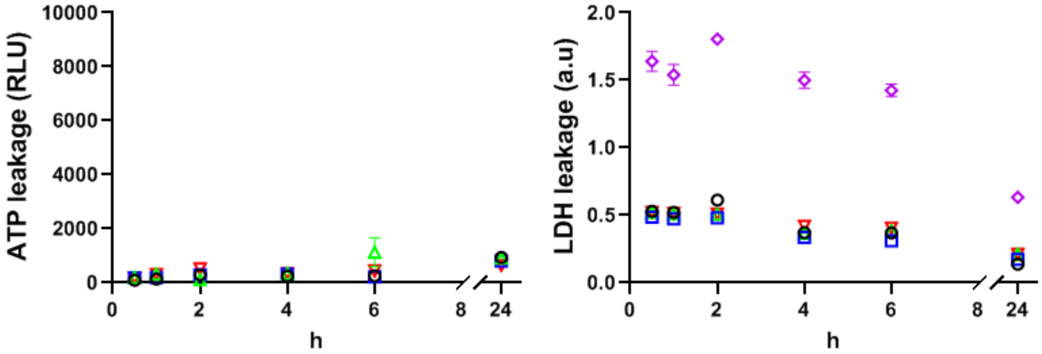

Figure S3. Quantification of ATP and LDH enzyme in the extracellular medium at different time points after MDCK1 cells incubation without light activation (blank symbols). ○ = control, Δ = Empty micelles, □ = Pheo alone, Δ = Pheo-micelles, ◊ = saponin positive control (detergent). Data are represented as mean ± Standard error of the mean. n=6.

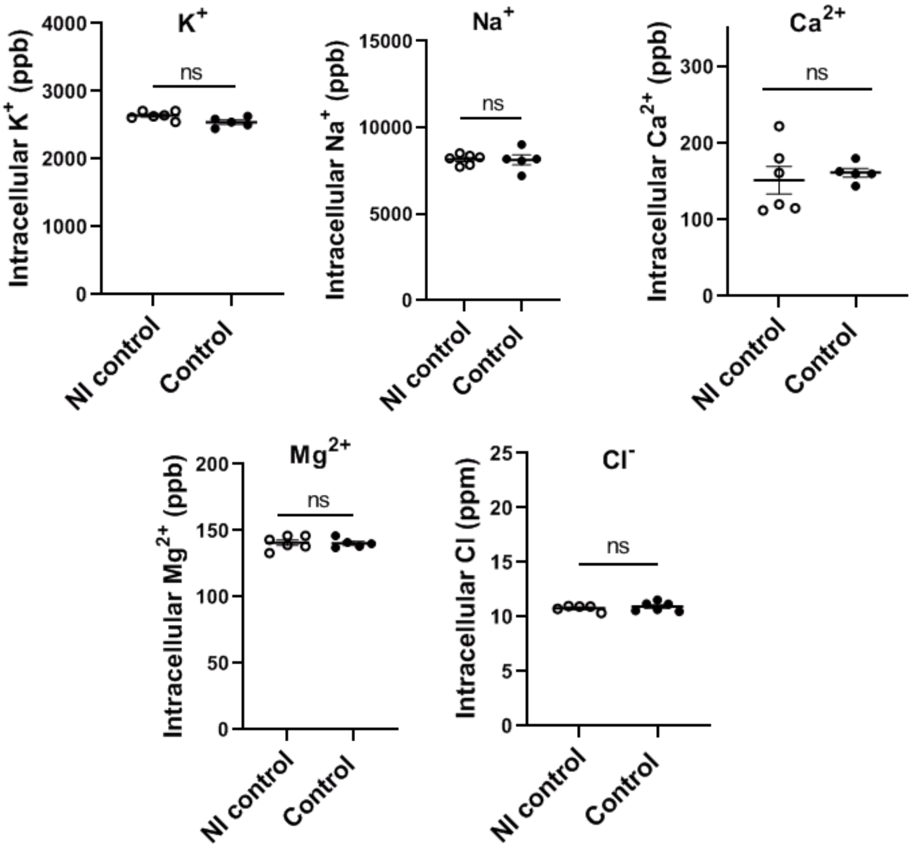

Figure S4. Ionic movements quantified by ICP-OES and ionic chromatography in non-irradiated (NI) and light irradiated MDCK1 cells. n=6. Data are represented as mean ± SEM

and analysed by 1-way ANOVA followed by Dunnett's multiple comparisons test which compare all conditions with the control condition. o = non-light irradiated (NI) control; ● = light irradiated control.

Attenuated total reflection. THz attenuated total reflection (THz-ATR) makes use of the evanescent wave at the back of a prism under total internal reflection,[41,42] which is coupled to the sample under study. When light is reflected at the interface of two optical media with the refractive index $n_1 > n_2$, total reflection occurs for $n_1 \sin\theta > n_2$, and an evanescent field exists in the less optically dense medium n_2 (**Figure S5**). When a sample is put on top of the prism, the evanescent wave extends into the sample. The measurement of the reflected beam is then directly correlated with the complex refractive index of the medium topping the prism. The penetration depth of the evanescent field depends on the beam wavelength and on the refractive index of the prism and of the topping medium. The penetration depth of the evanescent field depends on the beam wavelength and on the dielectric constants of the prism and of the topping medium. More precisely, we consider a thin layer of cells and the surrounding solution in this inhomogeneous evanescent wave. The stronger coupling is obtained for a penetration depth of the same value as the cell layer thickness. The resulting reflected THz signal is then correlated with the cytosol content value and repartition in the layer. The extracellular solution above the cell layer only weakly affects the THz signal because its volume is much larger than the intracellular one. Therefore, cytosol leakage from the cell to the surrounding solution can easily be detected by the change of the cell content.[10,11,45]

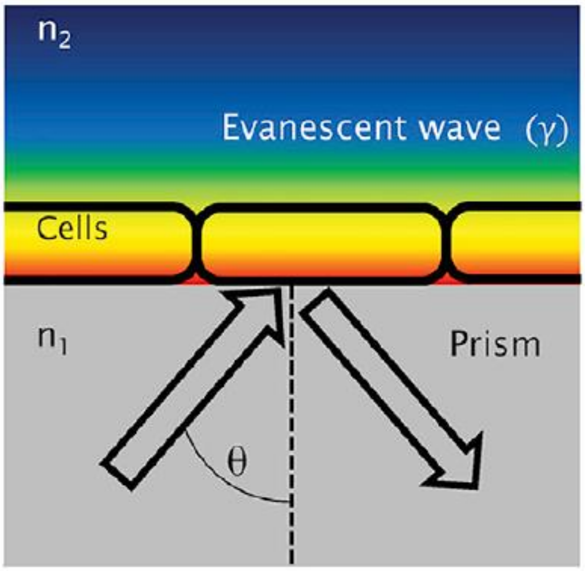

Figure S5. Close view of the ATR prism is shown. The cell layer lies in the evanescent field zone (in colour gradient).

THz-ATR illumination artefact. During the illumination phase of the cells, an artefact may be observed (**Figure S6**-Left). This artefact is not due to the actual cell response, but is related to the creation of positive and negative carriers in the semiconductor cell support. Since the presence of these carriers changes the conductivity of the semiconductor,[48] the THz-ATR reflection coefficient is modified accordingly. Because the carrier lifetime is much shorter than a microsecond, and thus much shorter than the time resolution of the experiment, the illumination creates an instantaneous alteration of the THz-ATR signal over the reference and cell areas. The recorded signal S_{THz} is the ratio of measurements in these two areas. Under experimental conditions, there is always a slight illumination imbalance between the two zones, leading to either a positive or negative illumination artefact. However, this artefact can easily be eliminated since it corresponds to a constant jump only during illumination. It is therefore easily recognizable. The artefact observed in **Figure S6**-Left was removed from the corresponding plots in **Figure S6**-Right.

THz-ATR control experiments. The average (data in parenthesis) of the normalized THz-ATR signals at concentration $C = 1.65\ \mu M$ is presented in **Figure S6**-Right for all conditions, with and without illumination. No effect is observed in the controls without illumination. With illumination, variation is only observed for Pheo alone and encapsulated Pheo.

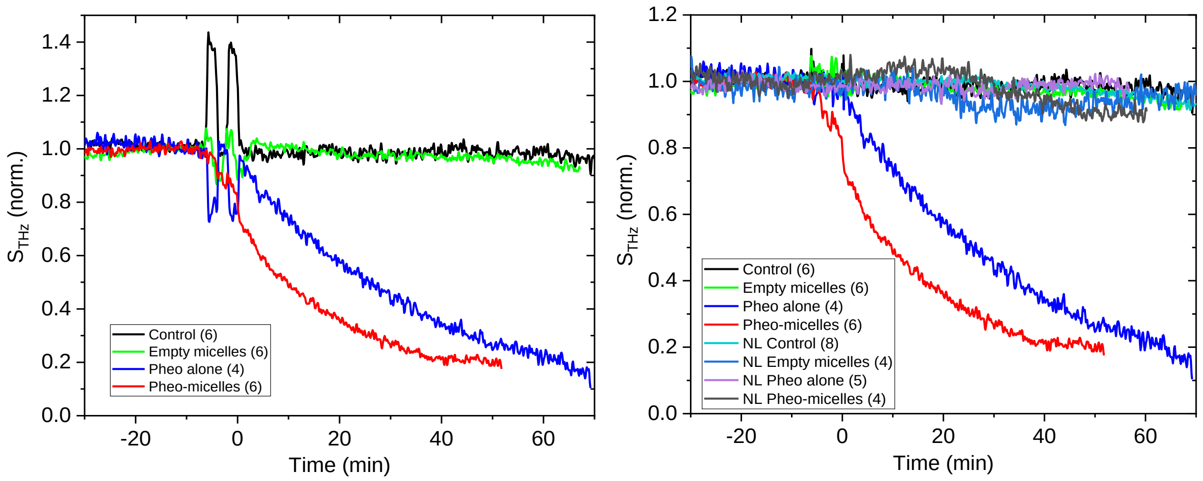

Figure S6. (Left) Direct normalized THz-ATR measurement. An illumination artefact is visible. (Right) Summary of all conditions after artefact correction. Mean values. M: micelles; P: Pheophorbide; L: light. Concentrations for PL and MPL are 1.65 µM.

THz-ATR parameter extraction. A typical recorded THz-ATR signal is outlined in **Figure S7**. Such a signal results from the ratio between the cell signal and the reference signal, in order to cancel out

the fluctuations of the THz laser and achieve optimal sensitivity and stability. After illumination at time $t = 0$ and stabilization of the signal for long delays, the cells are scraped off the support. This corresponds to the baseline of the measurement. Then, the normalized signal is obtained attributing the value 1 before illumination, and 0 after scraping. Finally, the normalized signal is fitted with an exponential function with time decay T and amplitude D. The parameter T provides the time scale of the evolution of the THz-ATR signal, and parameter D the magnitude of the variation of signal. A value $\Delta = 0$ means no variation at all after illumination. A value $\Delta = 1$ is the maximum possible.

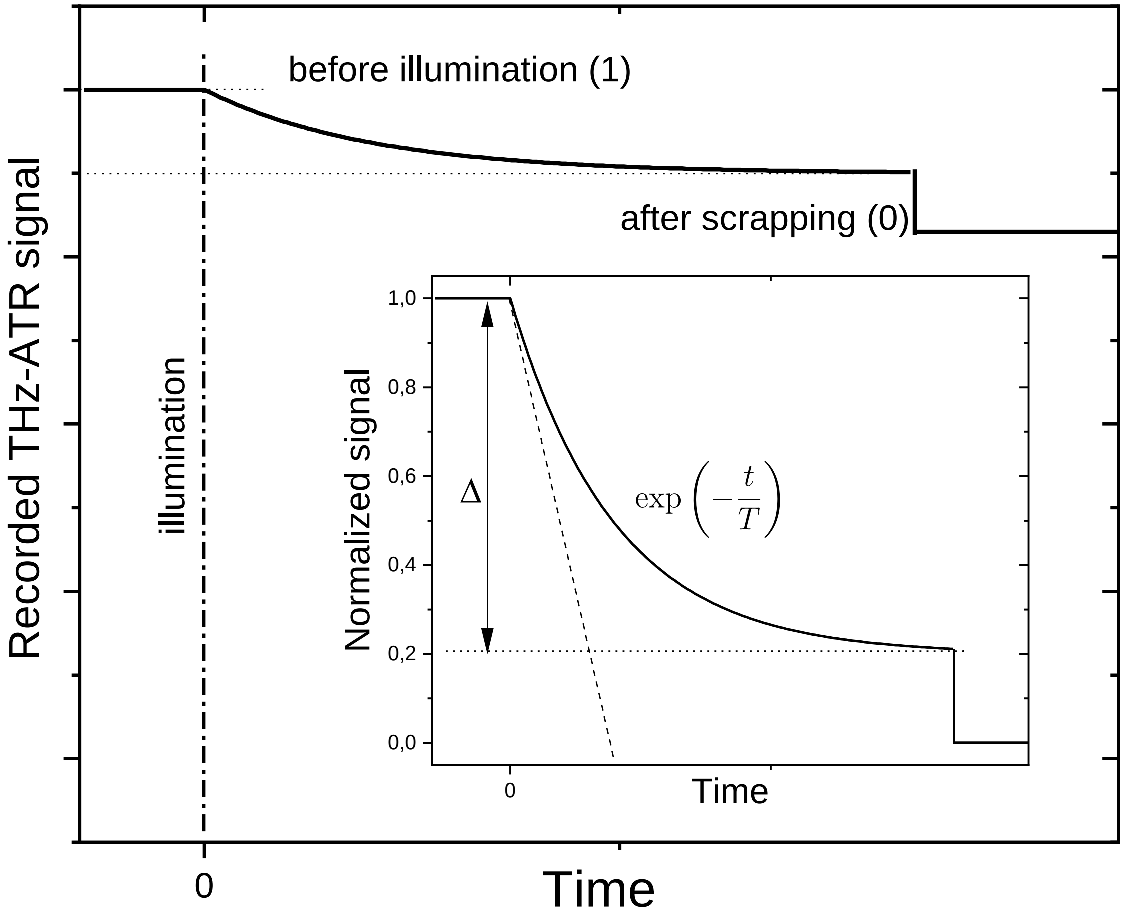

Figure S7. Parameters of the exponential fit of the THz signals. The main graph presents the recorded THz-ATR signal with illumination at time $t = 0$. After the signal is stabilized for lengthy periods, the cells are detached from the holder surface. The normalized signal is obtained taking a value of 1 before illumination, and 0 after scraping the cells. The data are then fitted with an exponential function with amplitude Δ and decay time T.

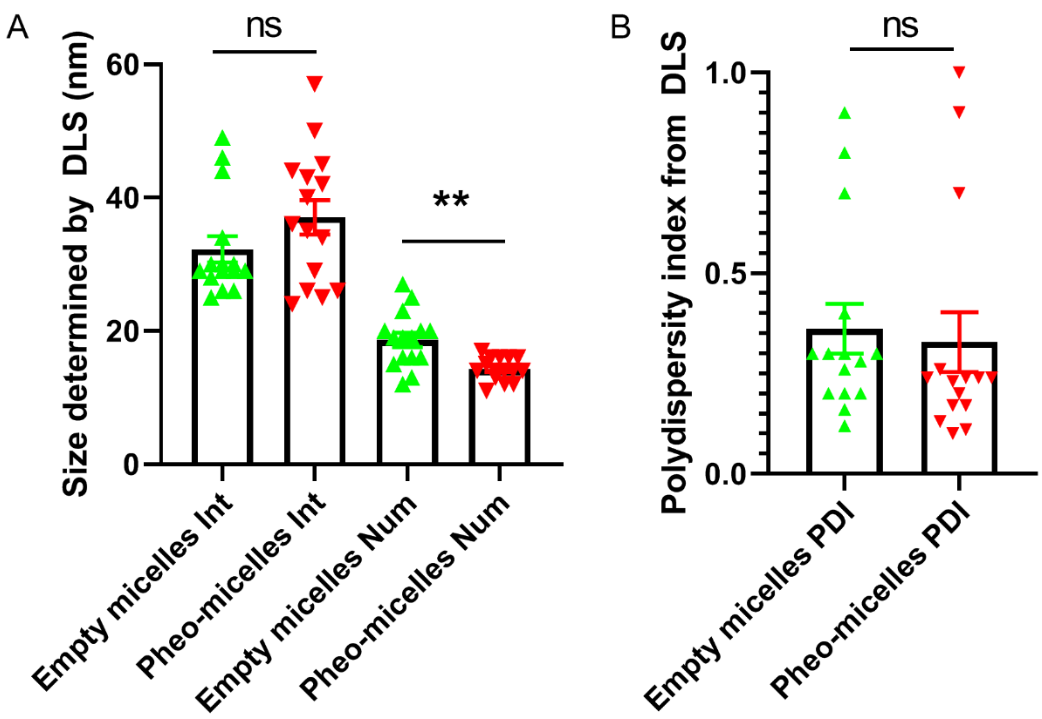

Figure S8. Characterization of polymeric micelles by Dynamic Light Scattering (DLS). A. determined size Int stands for intensity-average, Num for number-average. B. Polydispersity index. Data are represented as mean ± SEM. n=15.
